# Impact of thermal processing on phytochemical profile and cardiovascular protection of *Beta vulgaris* L. in hyperlipidemic rats

**DOI:** 10.1038/s41598-024-77860-2

**Published:** 2024-11-11

**Authors:** Engy Mohsen, Marwa I. Ezzat, Ibrahim E. Sallam, Dalia Zaafar, Aya Y. Gawish, Yasmine H. Ahmed, Ahmed H. Elghandour, Marwa Y. Issa

**Affiliations:** 1 Pharmacognosy Department, Faculty of Pharmacy, Cairo University , Kasr El-Aini Street, Cairo , 11562 Egypt; 2 Pharmacognosy Department, College of Pharmacy, October University for Modern Sciences and Arts (MSA) , 6th of October City, Giza 12566 Egypt; 3 Pharmacology and Toxicology Department, Faculty of Pharmacy, Modern University for Technology and Information , Cairo, 11571 Egypt; 4 Cytology and Histology Department, Faculty of Vet. Medicine, Cairo University , Giza, 12211 Egypt; 5 Communication Department, Military Technical College , Cairo, Egypt

**Keywords:** Beetroot, Thermal processing, Steaming and boiling, UPLC-QTOF-MS/MS, Phenolics and betanins, Cardioprotective activity, Plant sciences, Cardiology

## Abstract

Beetroot (*Beta vulgaris* L.) is globally recognized for its outstanding color and flavor. It has been acknowledged for its therapeutic value since the ancient Romans. It is used to treat cardiovascular disorders. The therapeutic benefits of red beetroot are due to the substantial amounts of various bioactive metabolites, such as ascorbic acid, carotenoids, nitrates, phenolics, and betalains. However, the bioavailability and shelf life of these substances are significantly affected by the considerable variations in their processing methods among different countries. The longevity of the extracts is prolonged by employing well-established preservation techniques, such as boiling and steaming, which involve the application of heat. Our study aimed to analyze and compare the phytochemical composition of raw and heat processed beetroot using UPLC-QTOF-MS/MS. In addition, the study aimed to assess the effectiveness of processed beetroot in protecting against cardiovascular complications in a rat model of obesity induced by high-fat diet (HFD). UPLC-QTOF-MS/MS phytochemical profiling revealed the presence of 51 compounds, including organic acids, flavonoids, phenolics, betanins, and saponins. All the extracts demonstrated a significant decline in MDA, TNF- *α*, and IL-6 levels, suppressed the TGF-*β* expression, and restored the serum catalase level to normal. Among all the tested extracts, the steamed extract exhibited the slightest percentage change in body weight (10.2 ± 6.4) and effectively lowered the TNF-α level to normal levels. In contrast, the normal histological structure of heart muscle fibers was notably preserved in the cardiac sections of rats pretreated with steamed and boiled beetroot extracts. Additionally, mild caspase-3 immunoreactivity was observed in the cardiac muscles. The current study demonstrated that the steamed beetroot extract showed improved cardioprotective properties compared to the fresh and boiled extracts.

**Supplementary Information:**

The online version contains supplementary material available at 10.1038/s41598-024-77860-2.

## Introduction

Beetroot (*Beta vulgaris* L.) is a biennial herb that belongs to the Amaranthaceae family. Beetroots are valuable sources of dietary fiber, vitamins, minerals, and antioxidants^1^. In addition, it is commonly included in regular diets, either in its fresh form (as a salad or juice) or through various cooking methods such as roasting, boiling, or steaming. It is also a widely used food colorant in manufacturing processes^2,3^.

Red beetroot has been recognized for its therapeutic properties since ancient Roman times^4^. The red beetroot is cultivated in numerous countries and is commonly used in drinks, as a food colorant, and as a side dish^5^. It is utilized to treat cardiovascular disorders and has properties that render it effective as an anticancer agent, hemostatic agent, emmenagogue, carminative, and renal protection^6^.

Red beetroot therapeutic benefits are due to the significant amounts of several bioactive metabolites, such as ascorbic acid, carotenoids, nitrates, phenolics, and betalains. Among these compounds are natural pigments, such as betalains, from which a highly concentrated extract known as betanin can be derived^7,8^. Beetroot also contains phenolics and flavonoids, which are other types of phytochemicals. Biological studies have shown that beetroot extract possesses antioxidant and anti-inflammatory properties, making it a promising treatment option for arthritis, chronic inflammation, liver diseases, and cancer-related diseases^9^. Other benefits include anti-depressant, anti-microbial, anti-fungal, diuretic, expectorant, and carminative effects^10^. Beetroot comprises elevated amounts of inorganic nitrates associated with its cardiovascular health benefits. The beneficial effect of red beetroot as a supplement is attributed to its ability to reduce the average arterial pressure, which is achieved through the presence of elevated levels of nitrate and nitrite, as well as a high concentration of phenolics. These components work together in a synergistic manner when consumed simultaneously^11^.

Triterpenoid saponins are another group of natural compounds found in beetroot. Triterpenoid saponins are glycosides consisting of an aglycone covalently linked to one or more sugar units through an ester or ether glycosidic bond. The sugars mainly comprise pentoses, hexoses, uronic acids, and 6-deoxyhexoses^12^. Numerous traditional medicinal plants found globally contain saponins, which exhibit diverse biological effects such as allelopathic, anti-arthritic, antiviral, cytotoxic, antidiabetic, and cardioprotective activities^13,14^.

Food processing plays a crucial role in terms of nutrition, as it can affect antioxidant and nutrient content, bioavailability, and activity. Processing methods can result in nutrient loss, compound degradation, or beneficial transformations^15^. Andersson and his colleagues examined the steaming and boiling effects on the sensory profile, microstructure, and mechanical properties of beetroot, parsnip, and Jerusalem artichoke^16^. They determined that the most effective thermal process for enhancing sensory properties and dietary fiber composition depends on the root carbohydrate composition. This composition directly affects the integrity of the cell wall and the solubilization of cell constituents during thermal processing. Furthermore, the study compared the effects of various processing methods, including fresh, dried, processed juice, processed jam, boiled, pickled, and pureed samples, on the antioxidant properties of the samples. The fresh, dried, and pureed beetroots revealed the highest total phenolic, flavonoid, and antioxidant activities^17^.

Obesity has reached epidemic levels, and it is a primary contributor to global disability. It has long been recognized as a risk factor for the development of diabetes, insulin resistance, dyslipidemia, and different cardiovascular diseases^18–20^. It is hypothesized that it contributes to the development and progression of heart failure with either a conserved or reduced ejection fraction. Obesity is associated with a variety of hemodynamic changes, as well as metabolic, inflammatory, and neurohormonal variations, all of which have the potential to affect heart remodeling and function^21^.

Cardiovascular diseases pose a significant risk to the health and well-being of humans that may eventually lead to morbidity and mortality worldwide. Cardiac remodeling is the term used to describe any alteration in the heart size, shape, or morphology^22^. It is induced by modifications in the extracellular matrix (ECM) and the phenotype of cardiomyocytes. Fibrosis is characterized by the overproduction of ECM components, primarily collagen, by resident fibroblasts in the myocardium. This process can be further exacerbated by elevated transforming growth factor-β (TGF-β) levels. It may also result in cardiac architecture disruption, which can exacerbate rigidity, deteriorate systolic or diastolic function, and exacerbate arrhythmia^23^.

An obesity rat model was used to induce cardiac remodeling using an HFD as per the procedures used by Zaafar and colleagues^24^. The authors emphasized the increased expression of TGF-β as an indicator of fibrosis, oxidative stress, cardiac remodeling, and the enhanced proinflammatory and proapoptotic pathway following the administration of the HFD for four weeks.

A strategy for preventing cardiovascular diseases has been demonstrated to be effective by a diet high in vegetables and fruits containing high concentrations of established antioxidant and protective compounds^25–27^. Antioxidant metabolites, such as vitamin E, vitamin C, phenolics, and carotenoids, are extensively present in various plant sources and have biological activities that improve human health^28^.

There is a scarcity of research on the impact of steaming and boiling on beetroot, as well as the use of chromatographic analysis to identify differences between fresh and processed specimens. Therefore, ultra-high-performance liquid chromatography (UPLC/MS) and chemometric analysis were employed to characterize and compare the components of steamed and boiled beetroots with the fresh sample. This is the first time such a technique has been used to determine the alterations in the chemical profiles of processed beetroot. Additionally, the authors of this study examined the potential role of fresh, steamed, boiled, and red beetroot extracts in mitigating the detrimental effects of HFD on the heart.

## Materials and methods

### Chemicals and materials

Acetonitrile and LC-MS-grade deionized water were acquired from Merck (Darmstadt, Germany), while formic acid (ACS grade) was obtained from Sigma-Aldrich. Other chemicals, reagents, and materials used in the study were purchased from Sigma Chemical Co. (USA).

### Plant material and extracts

The roots of *B. vulgaris* were collected in February 2022 from the Horticulture Research Center, Ministry of Agriculture, Giza. The plant was identified by Mrs. Therese Labib, a consultant at the Ministry of.

Agriculture and the former director of El-Orman Botanic Garden. A specimen sample was kept at the Department of Pharmacognosy, Faculty of Pharmacy, Cairo University, under the code number 4-8-24 F.

Three kilograms of these roots were cleaned and divided into three equal portions. The first part, weighing 1 Kg, was grated into small pieces and mixed with methanol for a day. The roots underwent two additional macerations until no further extraction was achieved, and the methanol solution was subsequently filtered. The fresh beetroot extract was consolidated and concentrated under vacuum at a temperature not exceeding 40 ºC until complete drying, yielding 97 g of dry powder from the fresh beetroot extract.

The second part, weighing 1 kg, was cut into cubes (2 × 2 cm^3^) and placed in a stainless steel pot with the least amount of distilled water. The boiled root cubes were left to cool at the end of this process, then homogenized and macerated in methanol. The boiled beetroot extract was then prepared as previously described for the fresh extract to give 103 g of the dry powder of beetroot boiled extract^2^.

The third part (1 Kg) was cut into cubes (2 × 2 cm^3^) and placed in stainless-steel mesh tightly fixed over a pot that contained one and a half liters of boiling water and kept boiling for 45 min. while covered with a lid to be cooked on steam. Afterward, the steamed beetroot cubes were kept to cool, homogenized, and left macerated with methanol. The steamed beetroot extract was then prepared as previously described for the fresh extract, resulting in 95 g of the dry powder of beetroot steamed extract^29^. The dry extracts were kept at − 20 °C for further research.

### Ultra-performance liquid chromatography-mass spectrometry analysis

The UPLC-QTOF-MS/MS system was equipped with a Waters Acquity binary solvent chromatographic pump and operated in Resolution Mode (M/ΔM ≥ 18,000). A reversed-phase Acquity BEH C18 column measuring 2.1 × 50 mm with 1.7-micron beads was utilized for a 3-minute separation, utilizing an eluent mixture of 0.1% formic acid in deionized water and 0.1% formic acid in acetonitrile. The chromatograms for High-Resolution Mass Spectrometry (HRMS) were generated using a Waters Synapt G2 hybrid Quadrupole-orthogonal acceleration time-of-flight configuration (Waters, Manchester, UK). The mass spectrometer was calibrated using sodium formate cluster ions and an orthogonal Lock-SprayTM. Leucine-enkephalin was utilized as a lock mass calibrant via the ESI probe for calibration purposes. The internal mass correction calibrant was the pseudo molecular leucine-enkephalin ion at *m/z* = 554.2615.

### Chemometric comparative study

Plausible correlations among the samples were investigated by subjecting chemical constituents to PCA (Principal Component Analysis). This analysis involved 51 variables across nine specimens, resulting in 429 data points. The software Minitab 17 (Minitab Ltd., Coventry, UK) was utilized for the PCA, involving a covariance matrix. An Euclidean distance matrix was also calculated following the approach outlined in Sneath and Sokal (1973). Subsequently, two-dimensional plots, including score and loading plots, were generated using STATISTICA (StatSoft, Inc. 2003).

The PCA analysis revealed 51 chemical metabolites, and the resulting two-dimensional PCA biplots included fresh beetroot and processed specimens like steamed and boiled treatments. These biplots encompassed all identified compounds obtained via UPLC/ESI-MS and were used as input for the PCA constructed on a covariance matrix to compute eigenvalues.

### In vivo experiment

#### Animals

The male Swiss albino rats, weighing between 150 and 200 g, were procured from the Faculty of Medicine, Al Azhar University (Cairo, Egypt). Upon arrival, the rats were allocated into five groups and housed under standardized conditions with a temperature (23 ± 2 ºC), humidity (55 ± 1%), and a 12/12-hour light/dark cycle. Before commencing the experiment, animals were left to adapt for a period of one week.

All methods were carried out in accordance with relevant guidelines and regulations with the ARRIVE guidelines^30^ and reviewed and authorized by the Ethical Committee for the Care and Use of Laboratory Animals at the Faculty of Pharmacy, Cairo University [ID: MP (3119)].

#### Experimental design

The extracted samples were administered at a dosage of 250 mg/kg b.wt^31^. The HFD composition used for inducing cardiovascular changes was adopted from Zaafar and colleagues’ methodology with slight modification. The HFD was prepared by mixing 20% sucrose, 10% ghee (saturated fat from animal), and 12% cholesterol powder into a basal diet, and rats were fed with HFD for a period of 30 days^24^.

The rats were randomly assigned to five equal groups (*n* = 6) using the following grouping method:

- Normal group: Rats received oral distilled water (representing the vehicle) for six weeks.
- Positive control group: Rats received water for two weeks, followed by the administration of HFD for an additional four weeks.
- Fresh extract: Rats were orally administered fresh beetroot extract for two weeks, followed by the administration of HFD for an additional four weeks.
- Boiled extract: Rats were orally administered boiled beetroot extract for two weeks, followed by HFD for an additional four weeks.
- Steamed extract: Rats were orally administered steamed beetroot extract for two weeks, followed by the administration of HFD for an additional four weeks.

All rats in the experimental groups were weighed at the beginning of the study and at the termination of the study (by the end of week 6) to determine the percentage change in body weight (BWT) using the following formula^24^:

$$\% Change\;in\;body\;weight = [(Final\;BWT - Baseline\;BWT)/Baseline\;BWT] \times 100$$

#### Blood collection and sampling

After the final dosage administration, rats in all the studied groups were anesthetized with ketamine (50 mg/kg) and xylazine (10 mg/kg) mixture injected intraperitoneally, following the previously outlined procedure^32,33^. The rats were sacrificed by cervical dislocation one day after the final administration of HFD and the protective agents. Blood samples were collected from each rat through the eye orbital canthus in a clean test tube centrifuged at 2000xg for 15 min. In order to use the sera in the ELISA assays, the sera were collected and placed into sterile Eppendorf tubes after being separated. Subsequently, they were stored at a temperature of -20 °C. The hearts were quickly extracted from the rib cages, dissected, and fixed in phosphate-buffered formalin overnight. The samples underwent dehydration and xylene treatment and were subsequently embedded in paraffin. Afterward, 3 μm sections were created, deprived of paraffin, and stained with hematoxylin and eosin (H&E) for histopathological analysis, following the procedure outlined previously^34^. Additionally, the samples were subjected to immunohistochemistry.

#### Biochemical parameters

##### Oxidative stress investigation

Lipid peroxidation evaluation. The lipid peroxidation colorimetric/fluorometric assay kit (BioVision^®^, Catalog # K739-100, CA, USA) was used to assess oxidative stress by determining malondialdehyde (MDA) levels, the byproduct of lipid peroxidation. The samples reacted with thiobarbituric acid (TBA) to produce the MDA-TBA adduct and then were measured calorimetrically at 532 nm^35^.

Estimation of Catalase enzyme by ELISA. The catalase activity colorimetric/fluorometric assay kit (BioVision^®^, Catalog # K773-100, CA, USA) was used to estimate the antioxidant enzyme effect that catalyzes the breakdown of hydrogen peroxide (H_2_O_2_) to water and oxygen. Firstly, catalase reacts with H_2_O_2,_ producing water and oxygen. Then, the unreacted H_2_O_2_ reacts with the OxiRedTM probe, resulting in a product that can be measurable at 570 nm^35^.

##### Assay of inflammatory markers in serum using ELISA

Estimation of Interleukin-6 levels by ELISA. A multifunctional cytokine called interleukin-6 (IL-6) controls various biological processes, such as acute-phase reactions, inflammation, and immune responses. BioVision^®^ ELISA kit (Catalog # 4145 − 100, CA, USA) was used to estimate the serum IL-6 levels. The reaction color changes from blue to yellow and the microwell absorbance is estimated at 450 nm using a microplate reader.

Estimation of Tumor necrosis factor-α levels using ELISA. Tumor necrosis factor-α is a potent multifunctional cytokine with regulatory, inflammatory, and cytotoxic effects on various cells. BioLegend’s ELISA MAXTM Deluxe Set (Catalog # 438204, San Diego, CA), a sandwich Enzyme-Linked assay kit, was used to assess serum TNF- levels (ELISA). The reaction color changed from blue to yellow, and the microwell absorbance was evaluated at 450 nm using a microplate reader.

##### Assessment of levels of Transforming growth factor beta (TGF-β) in serum via western blotting analysis

The heart tissues were homogenized in lysis buffer to extract proteins. The resulting homogenates were centrifuged, and the supernatants were gathered to determine protein level using the BCA assay. Subsequently, the cells were lysed by adding sodium dodecyl sulfate (SDS) buffer obtained from TCI CO., LTD, Japan. SDS-PAGE gel electrophoresis was carried out. The primary antibodies, diluted in TBST (1:500), were incubated overnight against the targeted protein on the blot, followed by a 1-hour incubation at ambient temperature with an HRP-conjugated secondary antibody solution (Goat anti-rabbit IgG- HRP-1 mg Goat mab -Novus Biologicals). The blot was rinsed 3–5 times with TBST for 5 min each. Finally, the chemiluminescent substrate (Clarity TM Western ECL substrate, Bio-Rad cat#170–5060) was applied to the blot according to the manufacturer’s instructions.

#### Histopathological examination

##### Light microscopy

Rats were anesthetized at the end of the experiment. The heart was quickly extracted and then fixed with 10% neutral buffered formalin. The fixed samples underwent dehydration, followed by xylene treatment, and were then embedded in paraffin. Sections with a thickness of 3 μm were prepared, deparaffinized, and stained with hematoxylin and eosin (H&E) for histopathological examination^36^.

##### Histopathological scoring

Rat myocardial injuries were assessed using the grading system developed by Atkinson and colleagues, allowing the classification of findings into distinct levels that represent varying degrees of histologic myocardial damage: edema, myocardial necrosis, hemorrhage, and inflammatory cell infiltrates using scores from 0 to 3. (0) no harmful impact, (1) mild injury, (2) slightly greater destruction, and (3) the most severe damage. This method was employed to determine the heart histopathological index (Atkinson et al., 2010).

##### Immunohistochemical investigation

Caspase-3, as a proapoptotic marker, was applied. The technique was used as described earlier^37^. Tissue sections treated with anti-Caspase-3 were examined using a digital Leica Quin 500 image analysis system located at the Faculty of Dentistry, Cairo University.

#### Statistical investigation

Data were displayed as mean values ± standard deviation. Paired Student t-tests were used for within-group comparisons, while between-group comparisons were conducted using one-way ANOVA, followed by Tukey’s post hoc test. Statistical analysis was performed using the SPSS Software, version 17 (SPSS Inc., Chicago, USA). The level of statistical significance was set at *P* < 0.05.

## Results

### Metabolite identification using UPLC-QTOF-MS/MS analysis

**Fig. 1:**
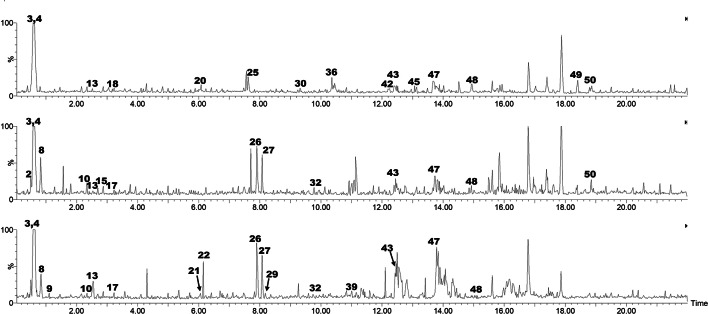
Base peak chromatograms from UPLC-ESI-MS of methanolic extracts of beetroot fresh (**A**), steamed (**B**), and boiled (**C**) samples. The peak numbers on the chromatogram correspond to those listed in Table 1.

The metabolites in the examined samples were detected using UPLC-QTOF-MS/MS. Three major metabolite classes were identified, including phenolics and organic acids, betanins and saponins. Table 1 presents the mass spectra data for the **51** identified metabolites. Metabolites were tentatively identified by UPLC/MS in fresh, steamed, and boiled beetroot extracts. The negative network clustered the related phenolics and saponins using MS/MS data. The abundance was observed in the elution section of the Retention time (0.53–14.92 min) (Table 1 and Fig. 1).

**Table 1:** Bioactive secondary metabolites putatively identified in fresh and processed beetroot extracts using UPLC-QTOF-MS/MS analysis.

Peak no.	RT Min.	[M-H]^−^	Fragments MS product ion	Tentative identification	Molecular formula	Fresh	Streamed	Boiled	Reference
Phenolics and organic acids									
1	**0.53**	145.0620	119, 101	**Methyl glutaric acid**	C_6_H_10_O_4_	+	-	-	^38^
2	**0.54**	215.0352	193, 171	**Bergapten**	C_12_H_8_O_4_	-	+	-	^39^
3	**0.57**	377.0856	341, 215, 179	**Caffeic acid derivative**	C_18_H_18_O_9_	+	+	+	^40^
4	**0.59**	719.1969	359, 197, 179, 161, 135	**Sagerinic acid**	C_36_H_32_O_16_	+	+	+	^41^
5	**0.68**	341.1096	179, 161, 143, 119, 101	**Caffeyol hexoside**	C_15_H_18_O_9_	+	+	+	^40^
6	**0.69**	133.011	97	**Malic acid**	C_4_H_6_O_5_	-	-	+	^42^
7	**0.78**	191.0181	173,111,87,129, 85	**(Iso)citric acid**	C_6_H_8_O_7_	+	+	+	^43^
8	**0.82**	128.0339	84	**Pyroglutamic acid**	C_5_H_7_NO_3_	-	+	+	^38^
9	**1.12**	577.1381	289, 287	**Procyanidin**	C_30_H_26_O_12_	+	+	+	^44^
10	**2.17**	505.4515	343, 297	**Decarboxy-betanin/isobetanin isomer1**	C_23_H_26_N_2_O_11_	-	+	+	^40,45^
11	**2.2**	343.0626	169, 125	**Galloylquinic acid**	C_14_H_16_O_10_	-	+	+	^42^
12	**2.41**	505.1429	343, 297	**Decarboxy-betanin/isobetanin isomer 2**	C_23_H_26_N_2_O_11_	-	+	+	^40,45^
13	**2.54**	359.0997	299, 229, 197, 153, 145	**Syringic acid -** ***O*** **- hexoside**	C_15_H_20_O_10_	+	+	+	^46^
14	**2.66**	233.1199	173, 125	**Acetyl quinic acid**	C_9_H_14_O_7_	-	-	+	^42^
15	**2.87**	305.0676	179,167,137,125	**(Epi)gallocatechin**	C_15_H_14_O_7_	-	+	-	^40^
16	**3.01.**	461.4451	301	**2**,**17-bidecarboxy-betanin/isobetanin**	C_22_H_26_N_2_O_9_	-	+	+	^40,45^
17	**3.12**	577.1383	289, 287	**Procyanidin isomer**	C_30_H_26_O_12_	+	+	+	^44^
18	**3.17**	307.0995	289, 249, 205	**Catechin hydrate**	C_15_H_16_O_7_	+	-	-	^40^
19	**5.61**	137.0087	93	**Hydroxybenzoic acid**	C_7_H_6_O3	+	+	+	^40,46^
20	**6.08**	153.0159	109, 79	**Dihydroxybenzoic acid**	C_7_H_6_O_4_	+	-	-	^40,46^
21	**6.11**	355.101	193, 178	**Ferulic acid hexoside**	C_16_H_20_O_9_	+	+		^45,47^
22	**6.15**	135.043	120, 91	**Methyl benzoic acid**	C_8_H_8_O_2_	-	-	+	^43,46^
25	**7.6**	329.231	311, 293, 211, 197, 149, 113	**Trihydroxy octadecenoic acid**	C_18_H_33_O_5_	+	-	-	^46^
28	**8.13**	137.0092	93	**Hydroxybenzoic acid isomer**	C_7_H_6_O_3_	-	+	+	^43,46^
31	**9.68**	663.1461	545, 311	**Malonyl 6’’-** ***O*** **-deoxyhexosyl -C-hexosyl apigenin**	C_30_H_32_O_17_	+	+	-	^46^
32	**9.73**	215.0317	199, 185, 171	**Bergapten isomer**	C_12_H_8_O_4_	-	+	+	^39^
33	**9.84**	433.1126	-	**Apigenin-7-** ***O*** **-glucoside**	C_21_H_20_O_10_	+	+	-	^44^
35	**10.19**	313.24	251.22	**Octadecanedioic acid**	C_18_H_34_O_4_	+	-	-	^46^
39	**10.94**	315.2527	297.24, 253.03	**Dihydroxyoctadecanoic acid**	C_18_H_36_O_4_	-	-	+	^46^
40	**11.43**	297.1495	163.04, 107.04	**Auraptene**	C_19_H_22_O_3_	-	-	+	^11^
41	**11.95**	325.1871	163, 119	**Coumaroylhexose**	C_15_H_18_O_8_	+	+	+	^42^
42	**12.21**	279.2291	217.24	**Linoleic acid**	C_18_H_32_O_2_	+	-	-	^48^
43	**12.45**	311.1696	243, 183, 145, 119, 99, 53	**Dihydroxyoctadecadienoic acid**	C_18_H_32_O_4_	+	+	+	^46^
44	**12.99**	325.1811	163, 119	**Coumaroylhexose isomer 1**	C_15_H_18_O_8_	+	+	+	^42^
45	**13.06**	299.2574	281, 255	**2-Hydroxyoctadecanoic acid**	C_18_H_36_O_3_	+	-	-	^46^
46	**13.25**	353.1997	191,179,135	**Caffeoylquinic acid**	C_16_H_18_O_9_	+	-	+	^42^
47	**13.79**	325.187	163, 119	**Coumaroylhexose isomer 2**	C_15_H_18_O_8_	+	+	+	^42^
48	**14.92**	279.2294	217.24	**Linoleic acid isomer**	C_18_H_32_O_2_	+	+	-	^48^
Saponins									
23	**6.35**	1087.4811	967, 925, 763, 743, 593, 455	**Act-Pen-Hex-UrA-oleanolic acid**	C_52_H_79_O_24_	+	+	+	^49^
24	**7.5**	1087.5156	955, 793, 745, 455	**Hex-Pen-Hex-UrA-oleanolic acid**	C_53_H_83_O_23_	-	+	+	^49^
26	**7.9**	925.4801	745, 569, 551	**Hex-Pen-UrA-oleanolic acid**	C_46_H_69_O_19_	-	+	+	^50^
27	**8.08**	793.9479	631, 569, 455	**Hex-Hex-UrA-oleanolic acid**	C_42_H_65_O_14_	-	+	+	^12^
29	**8.18**	955.4405	835, 793, 631, 455	**Act-Hex-UrA-oleanolic acid**	C_47_H_71_O_20_	-	+	+	^51^
30	**9.31**	925.4755	745, 569, 551	**Hex-Pen-UrA-oleanolic acid isomer**	C_46_H_69_O_19_	+	+	-	^50^
34	**10.1**	763.4282	631, 455	**Pen-UrA-oleanolic acid**	C_41_H_63_O_13_	+	+	+	^50^
36	**10.36**	793.9441	673, 631, 455	**Hex-Hex-UrA-oleanolic acid isomer**	C_41_H_61_O_15_	+	-	-	^49,50^
37	**10.59**	791.3728	631, 455	**Diox-UrA-oleanolic acid**	C_41_H_59_O_15_	+	-	-	^50^
38	**10.75**	791.3828	631, 455	**Diox-UrA-oleanolic acid isomer**	C_41_H_59_O_15_	+	-	-	^50^
49	**18.41**	763.558	631, 455	**Pen-UrA-oleanolic acid isomer**	C_41_H_63_O_13_	+	+	+	^50^
50	**18.84**	807.5686	647, 471	**Act-UrA-gypsogenin**	C_41_H_59_O_16_	+	+	+	^50^
51	**21.7**	809.3899	689, 647, 513, 471	**Act-UrA-hederagenin**	C_41_H_61_O_16_	+	+	+	^49,50^

(+) and (-) indicate the presence and absence of the metabolites.

#### Phenolics and organic acids

The identified phytoconstituents consisted of 38 metabolites, primarily phenolics and organic acids. These metabolites were polar and were eluted by a solvent with a high proportion of water (Table 1). In addition, phenolic compounds were found to contain certain acids. **Peak 19**,** 20**,** and 22** correspond to hydroxybenzoic acid, dihydroxy benzoic acid, and methyl benzoate with *m/z* 137.0087, 153.0159 and 135.043 [M-H]^−^, respectively^40,46^.

**Peak 7** was the first detected organic acid with [M − H]^−^ at 191.018 *m/z* (C_6_H_8_O_7_), which yielding fragmentation ions at 173 *m/z* [M − H_2_O−H]^−^ and *m/z* 111 [M − 2H_2_O − COO − H]^−^. Consequently, it was identified as (iso)citric acid^43^. **Peak 3** showed [M − H]^−^ at *m/z* 341.177 (C_15_H_18_O_9_) as a typical caffeic acid hexoside, with product ion at 179 *m/z* [M − C_6_H_10_O_5_−H]^−^^40^. Ferulic acid hexoside was detected at **peak 21** with *m/z* 355.1 [M − H]^−^. The negative MS2 analysis revealed distinct fragment ions at m/z 193, which can be attributed to the loss of a hexosyl moiety [M − H−162]^−^^45,45^.

**peaks 41**, **44**, and **47** were found to have a peak ion at 163 m/z and 119 m/z, which can be attributed to the presence of coumaric acid. These peaks also showed a characteristic loss of a hexose unit, indicating that they can be labeled as coumaric acid hexosides. Coumaric acid exhibited both anti-inflammatory and antioxidant properties^42,52^.

Bergapten, a 5-methoxyfurocoumarin compound, was found in a steamed specimen at peak 2. This compound acts as a hepatoprotective agent and has anti-inflammatory and antioxidative properties^39^. Auraptene, **peak 40**, is a naturally occurring bioactive monoterpene coumarin with a potential therapeutic role as anti-inflammatory and antioxidant activities with an excellent safety profile^11^. A rosmarinic acid derivative, sagerinic acid, **peak 4** gave a prominent parent ion [M − H]^−^ at 719.1969 *m/z* and a base peak at 359 *m/z* corresponding to [M − 2 H]^2−^^45^.

The precursor ion observed at *m/z* 663.1461 [M-H]- in **peak 31** corresponded to the malonylated apigenin hexoside with an *O*-deoxyhexosyl moiety, as suggested by the MS^2^ spectra. This assertion was supported by specific daughter ions seen at *m/z* 545 [M − H−86 − 18]^−^, indicative of H_2_O loss, and at *m/z* 311 [M − H−146 − 120]^−^, confirming the loss of *O*-deoxyhexosyl residue. Additionally, cross-cleavages specific to 6’’-*O*-deoxyhexosyl *C*-hexosyl products were observed, that tentatively assigned the compound as malonyl 6’’-*O*-deoxyhexosyl *C*-hexosyl apigenin further supporting the concluded identification^46^.

Procyanidin isomers detected in this study at **peaks 9** and **17** are reported to provide natural chromogenic substances to beetroot^44^.

#### Betanins

We discovered some betanin breakdown products, viz., 17-decarboxy-betanin and 2, 17-bidecarboxy-betanin. Those composites with *m/z* values of 505 [M − H]^−^ and 475.1 [M − H]^−^, respectively, correspond to **peaks 10**, **12** and **16**. It has been reported that high temperatures strengthen the occurrence of decarboxylation and oxidation of betacyanins and produce betacyanin derivatives^40^.

**Peak 12** showed [M − H] − at m/z 505.1429, which was broken to give 341, corresponding to *2*,17-bide carboxy-betanin/isobetanin^45^. **Peak 16** with *m/z* 461.2151 and fragment ion 301 matched decarboxy-betanin/isobetanin. These metabolites are the main byproducts of enzymatic oxidation that may be introduced during storage and heat processing but maintained under mild treatment^40,45^. Consequently, it was detected as characteristic of a steamed sample.

#### Saponins

Saponins were the most considerable class of the discovered compounds, represented by 13 metabolites. Their abundance is detected within Rt (6.35–21.7) min. elution region of Fig. 1. The structure of saponin glycosides possesses eminently similar chromatographic properties, proving numerous isomers. In red beetroots, structure similarity complicates sapogenin isomer separation and /or identification, which is considered a further challenge in the analysis of beetroot samples^12^.

Triterpene saponins comprise diverse structures in accordance with different aglycones, sugar moieties, and their uronic acid linkages. The mostly found sugar moieties in saponins of the investigated red beet (*B. vulgaris* L.) are monosaccharides viz., hexose (Hex.) and pentose (Pen.) units, and the replaced sugar residues of the acetal (Act.) and dioxolane (Diox.) type^12^.

In this study of beetroot, three triterpene aglycone moieties were detected with characteristic fragment ions viz., oleanolic acid, gypsogenin, and hederagenin acid at *m/z* 455.3525, 469.3318 and 471.3474, respectively^49^.

The smallest detected mass within all saponins were **peaks 34** and **49**, which possessed a precursor ion *m/z* 763.4016; its fragmentation pattern implied the occurrence of pentose and uronic acid (product ion *m/z* 631 [M–132–H]^–^) and 455 [M–132–176–H]^–^, respectively), hence they were identified as pentose-uronic acid-oleanolic acid and its isomer^50^.

**Peaks 27** and **36** corresponded to the identical precursor ions [M–H]^–^ at *m/z* 793.9479 and 793.9441, respectively. Their fragmentation resigned fragment ions at 631 and 455 *m/z* due to losses of 162 and 176 Da, indicating the occurrence of hexose, hexuronic acid, and oleanolic acid aglycone ions at *m/z* 455. These molecules comprised the same aglycone ion and sugar unit linked in different positions and recognized as Hex-HexUA-oleanolic acid^53^.

Saponins at **peak 29** with pseudomolecular ion [M–H]^–^ with *m/z* 955.4405 conveyed fragmentation ions at *m/z* 835, 793, 631 in accordance with the loss of deoxy-acetal moiety, hexose, and uronic acid and giving rise to abundant aglycone ion with *m/z* 455. Saponin **29** was a glycoside of oleanolic acid differing from **peak 30** by the existence of acetal instead of hexose. Hence, it is identified as Act-Hex-UrA-oleanolic acid^50^.

The **peaks 26** and **30** corresponded to the compound which exhibited the precursor ion [M-H]^–^ at *m/z* 925.4807 and 925.4805, respectively. Based on the fragmentation pattern, these metabolites comprised the same aglycone with *m/z* 455.352, which coincides with oleanolic acid, with the same compositions of sugars (i.e., pentose, hexose, and uronic acid) that were linked in two separate positions, Pen-HexUA-oleanolic acid^50^.

In addition, triterpene saponins with aglycones substituted with one or more sugar units, acetal or dioxolane-type substituents, give rise to a complex multicomponent mixture that distinguishes red beetroots extract.

**Peaks 37** and **38** with the molecular [M–H]^–^ ion at *m/z* 791.3728 and concerning MS/MS data with 160 Da loss confirmed the presence of an uncommon substituent in *Beta vulgaris* L. species. Those corresponded to dioxolane, creating a fragment of *m/z* 631, followed by loss of uronic acid residue. The cleavage of the dioxolane substituent formed the 160 Da fragment, indicating the fragmentation pattern of Diox-UrA-oleanolic acid^50^.

A different precursor ion [M-H]^–^ at *m/z* 807.5616 of **peak 50** with aglycone moiety characteristic to gypsogenin 469 [M − C_3_H_4_O_5_−C_2_H_2_O-UrA-H]^−^and 627 [M − C_3_H_4_O_5_−C_2_H_2_O−H−H]^−^, hence, identified as Act-UrA-gypsogenin^50^. Whereas **peak 51** with pseudomolecular ion [M-H]^–^ at *m/z* 809.3819 was identified as Act-UrA-hederagenin with the diagnostic fragment ions at *m/z* 471.3474, which resemble the aglycone moiety of hederagenin. The resulting fragments are 689 [M − C_3_H_4_O_5_H], 647 [M − Act − H], 513 [M − C_3_H_4_O_5_−UrA − H], and 471 [M − C_3_H_4_O_5_−C_2_H_2_O−UrA − H]. The presence of the acetal group in red beet saponins has previously been observed^12,50^.

Concerning **peak 23** at [M − H]^−^ with *m/z* 1087.4811 and fragmentation ions 967 (upon CID in negative ionization mode, cross-ring cleavages of the sugar moieties occurred), 925 (loss of acetyl group), 805 (cross-ring sugar moieties cleavages and loss of hexose), 763 (loss of hexose and acetyl group), 743 (cross-ring cleavages, loss of hexose and CO_2_^−^), 593 (743 − pentose) and finally 455 (oleanolic acid), gave the characteristic fragmentation pattern of Act- Pen - Hex − UrA − oleanolic acid^49^. **Peak 24** with molecular ion [M − H]^−^ with *m/z* 1087.5156 and fragment ions at 925 (loss of hexose), 793 (loss of hexose − pentose), 731 (loss of hexose − CO_2_ − pentose), 455 oleanolic acid was identified as Hex-Pen-Hex-UrA-oleanolic acid^49^.

#### Principal component analysis (PCA)

**Fig. 2:**
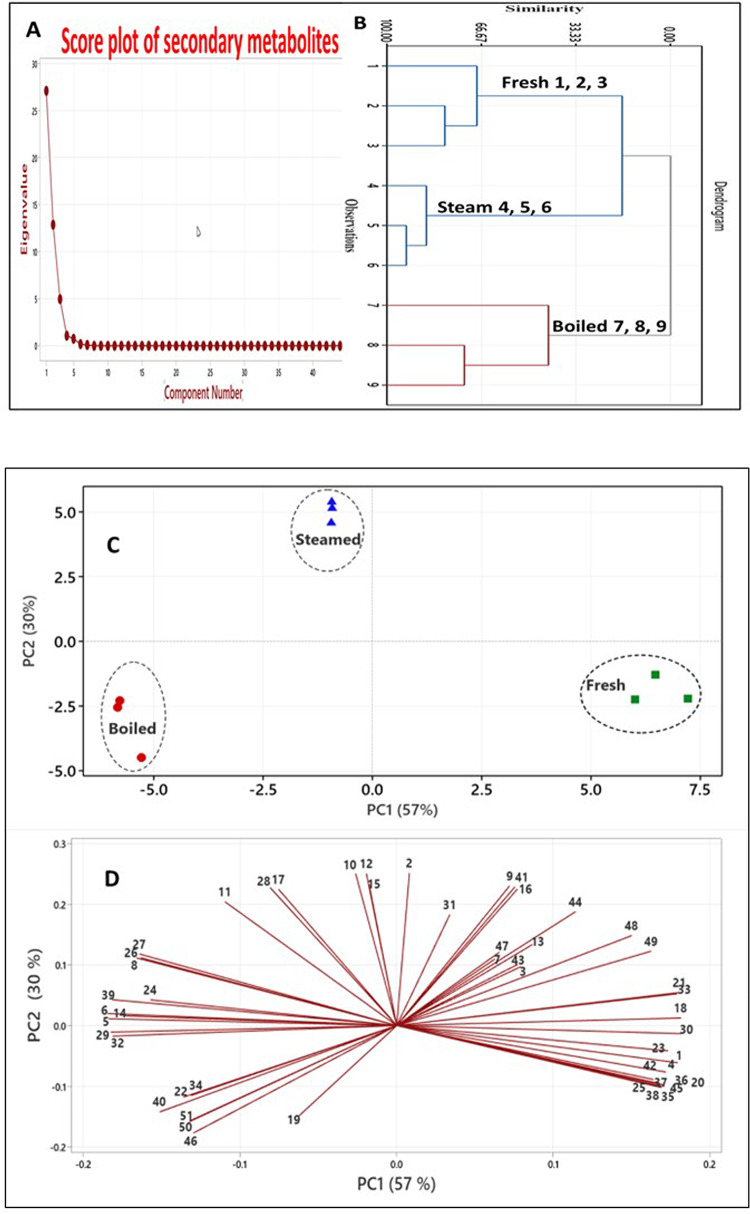
(**A**) Score plot of secondary metabolites according to their eigenvalues and (**B**) dendrogram representing similarity among specimens; fresh and processed samples of beetroot viz., steamed and boiled (**C**) PCA Score plot showing the arrangement of fresh and processed samples of beetroot viz., steamed and boiled (**D**) PCA Loading plot showing variable correlation within the matrix, PCA; principal component analysis.

PCA was performed to visualize the contribution of the phytoconstituents to the chemical profile of the three tested beetroot samples. This analysis highlighted differences in the secondary metabolites during heat processing. PCA facilitated the differentiation of the tested specimens. Figure 2A shows the score plot of secondary metabolites demonstrating their individual eigenvalues. Moreover, Fig. 2B displays a dendrogram that illustrates the similarity between different specimens of beetroot, including fresh and processed samples such as steamed and boiled. This similarity is determined based on the calculated eigenvalues.

PC1 and PC2 accounted for 87% of the total variance. The PCA score plot and loading plot, depicted in Fig. 2, demonstrate the relationships between beetroot specimens and their corresponding constituents. The data variability percentile was computed using the complete set of the first two principal components (PCs). PCA1 accounts for 57% of the variability, while PCA2 accounts for 30%. PCA graphs revealed the presence of three distinct groups, indicating a significant impact on the chemical composition of beetroot. The data inconsistency is mostly attributed to the variation of most saponin glycosides as diox-UrA-oleanolic acid and its isomer (values of eigenvectors: 0.17; -0.1 and 0.17; -0.09) and hex-Pen-UrA-oleanolic-acid isomer (0.18; 0.01), in addition to dihydroxyoctadecanoic acid (-0.18; 0.04) and Trihydroxy octadecenoic acid (0.17; -0.09) in the lower half at right side of the first PC, characteristic for fresh beetroot.

Then the following constituents are located on the middle of upper half of the loading plot, viz., betanins; decarboxy-betanin/isobetanin (values of eigenvectors: -0.03; 0.25) and 2,17-bidecarboxy- -betanin/isobetanin (0.07; 0.23), followed by (Epi) gallocatechin (-0.02; 0.24), procyanidin isomer (-0.08; 0.23) and Hydroxybenzoic-acid isomer (-0.09; 0.23) characterizing the steamed beetroot in the second PC. Finally, the boiled samples were in the lower half of the left side of the score plot. They were mainly interrelated by a relatively high content of auraptene (values of eigenvectors: -0.15; -0.15), act-UrA-gypsogenin (-0.13; -0.16), and caffeoylquinic acid (-0.13; -0.19). The components lying on the upper right half of Fig. 2 exhibit a significant similarity between fresh and steamed specimens, suggesting that the availability of compounds remains unchanged during this type of processing. This was mediated through various componoents such as the three coumaroylhexose isomer, dihydroxyoctadecadienoic acid, syringic acid *O*- hexoside, caffeic acid derivative, ferulic acid hexoside, Hex-Pen-UrA-oleanolic acid. The presence of a majority of glycosidic forms of phenolic acids or saponins in the group of constituents suggests that steam treatment of beetroot effectively retains the phytoconstituents in their original state and largely preserves their chemical composition, as opposed to boiling.

### Impact of various beetroot extracts on the body weight of rats consuming a high-fat diet

By the end of week six, all experimental groups demonstrated a significant increase in final body weight compared to their initial measurements. Notably, the final body weight of rats in the positive control and fresh beetroot groups was markedly higher than that of the normal group. Assessing the percent change in body weight at the end of the study revealed a notably higher percentage in the positive control group rats compared to all other tested groups (38.8 ± 5.7 vs. 4.8 ± 2.3, 19.6 ± 12.3, 19.4 ± 7.9, and 10.2 ± 6.4, respectively), as depicted in Table 2.

**Table 2:** Impact of various beetroot extracts on the body weight of rats consuming a high-fat diet.

Groups	Baseline b. wt. (g)	Final b. wt. (g)	% change in b. wt.
Normal control	211 ± 9.6	221.2 ± 13.7*	4.8 ± 2.3
Positive control	214 ± 4.2	297 ± 14.4*#	38.8 ± 5.7
Fresh extract	212 ± 13.4	252.6 ± 15.8*#▲	19.2 ± 12.3
Boiled extract	198.8 ± 10.8	237.2 ± 18.9*▲	19.3 ± 7.9
Steam extract	213 ± 10.95	233.8 ± 15.9*▲	9.8 ± 6.4

The results are presented as mean ± SD. The paired T-test assessed changes in data within each group between baseline and final body weight. One-way ANOVA was used to analyze data among groups, followed by Tukey’s post hoc test to determine significance at *p* < 0.05. * Indicates significance compared to the baseline body weight of the same group. # Indicates significance compared to the normal group. ▲ Indicates significance compared to the positive control group. B. wt. Refers to body weight.

### Impact of various beetroot extracts on lipid peroxidation through MDA release estimation

**Fig. 3:**
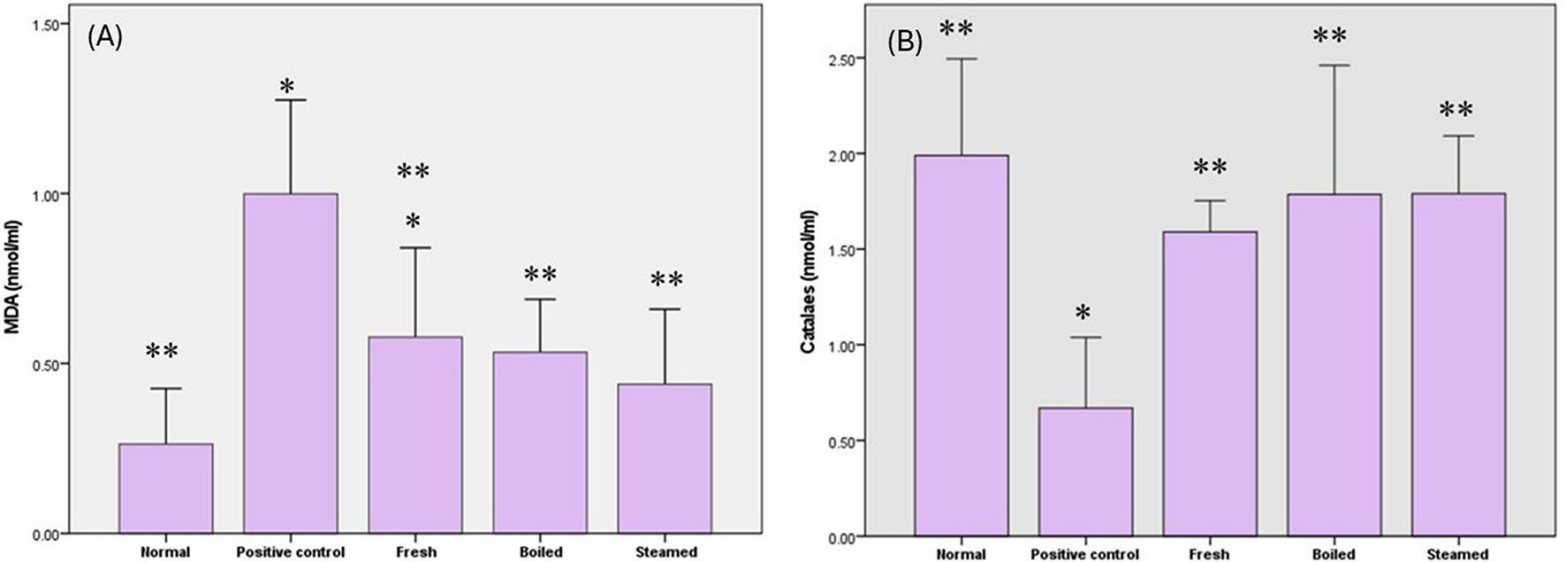
The impact of beetroot extracts on (**a**) malondialdehyde (MDA) and (**b**) catalase levels in high-fat diet-fed rats. The data are presented as mean values with standard deviation (SD). The analysis involved one-way ANOVA with Tukey’s post hoc test. * indicates statistically significant differences between groups at *P* < 0.05.

The current findings highlighted the significant elevation of MDA levels in rats fed with an HFD by 280% compared to rats on a normal diet. All pretreated beetroot extracts combined with an HFD showed a significant decline in MDA level of 42% in fresh extract, 46.6% in boiled extract, and 56% in steam extract compared to the positive control group. The boiled and steamed extracts successfully normalized MDA serum levels (Fig. 3a).

### Impact of various beetroot extracts on oxidative stress by estimation of catalase level

The serum level of catalase decreased significantly in rats fed with an HFD by 66.3% compared to rats fed a normal diet. However, the level of catalase enzyme in the sera of rats pretreated beetroot extracts returned to normal levels (Fig. 3b).

### Impact of various beetroot extracts on the inflammatory status of different beetroot extracts

**Fig. 4:**
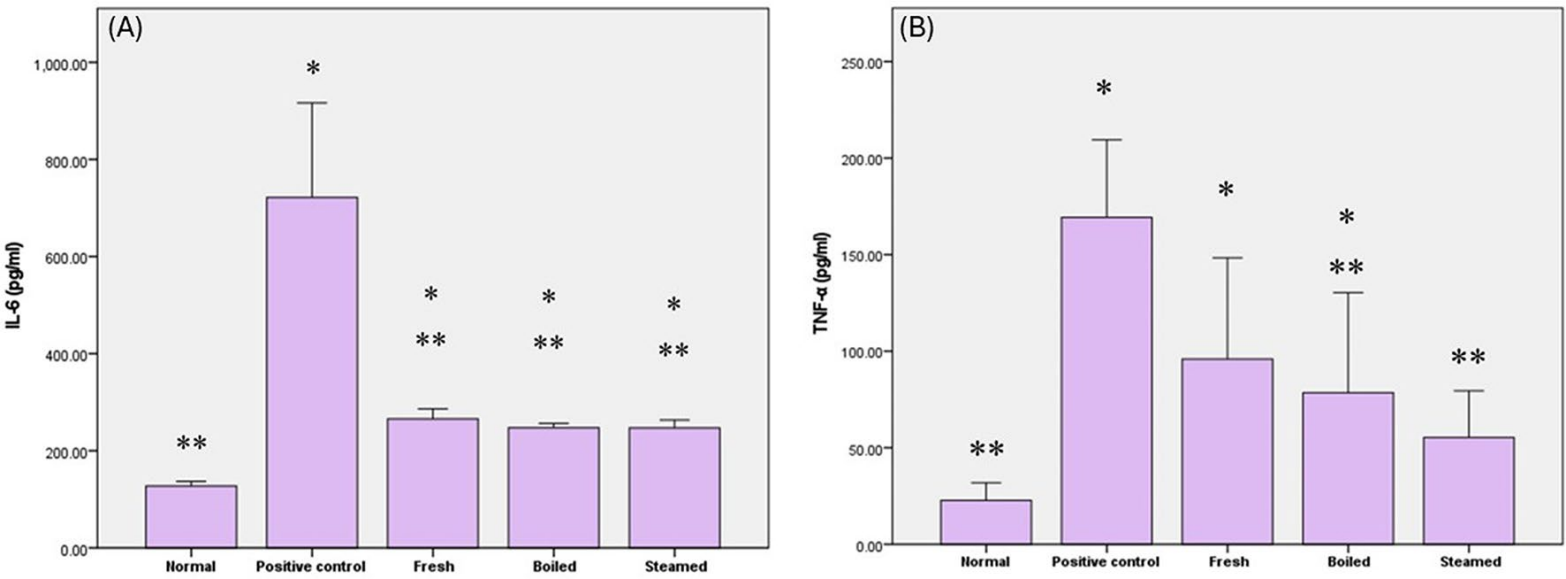
The impact of beetroot extracts on (**a**) Tumor Necrosis factor alpha (TNF-α) and (**b**) Interleukin-6 (IL-6) levels in rats subjected to a high-fat diet. The data are presented as mean values accompanied by standard deviation (SD). The analysis was conducted using one-way ANOVA and Tukey’s post hoc test. * indicates statistically significant differences between groups at *P* < 0.05.

The group of rats fed with the HFD demonstrated a high inflammatory response and a significant increase in both TNF- *α* and IL-6 biomarkers by (642.6% and 466%, respectively) compared to normal, whereas rats pretreated with beetroot extracts exhibited a significant decrease in TNF- *α* and IL-6 compared to the positive control group by (43.3% and 63% in fresh group, 53.6% and 65.6% in boiled extract group) respectively and 65.7% in steamed extract group for IL-6. Moreover, the pretreated steamed extract successfully showed normalized levels of TNF- *α.* The pretreated three beetroot extracts exhibited a statistically significant reduction in IL-6 compared to the normal group by 108.3% for fresh extract, 94% for boiled extract, and 93.7% for steamed extract group (Fig. 4).

### Impact of various beetroot extracts on the expression of TGF-*β*

**Fig. 5:**
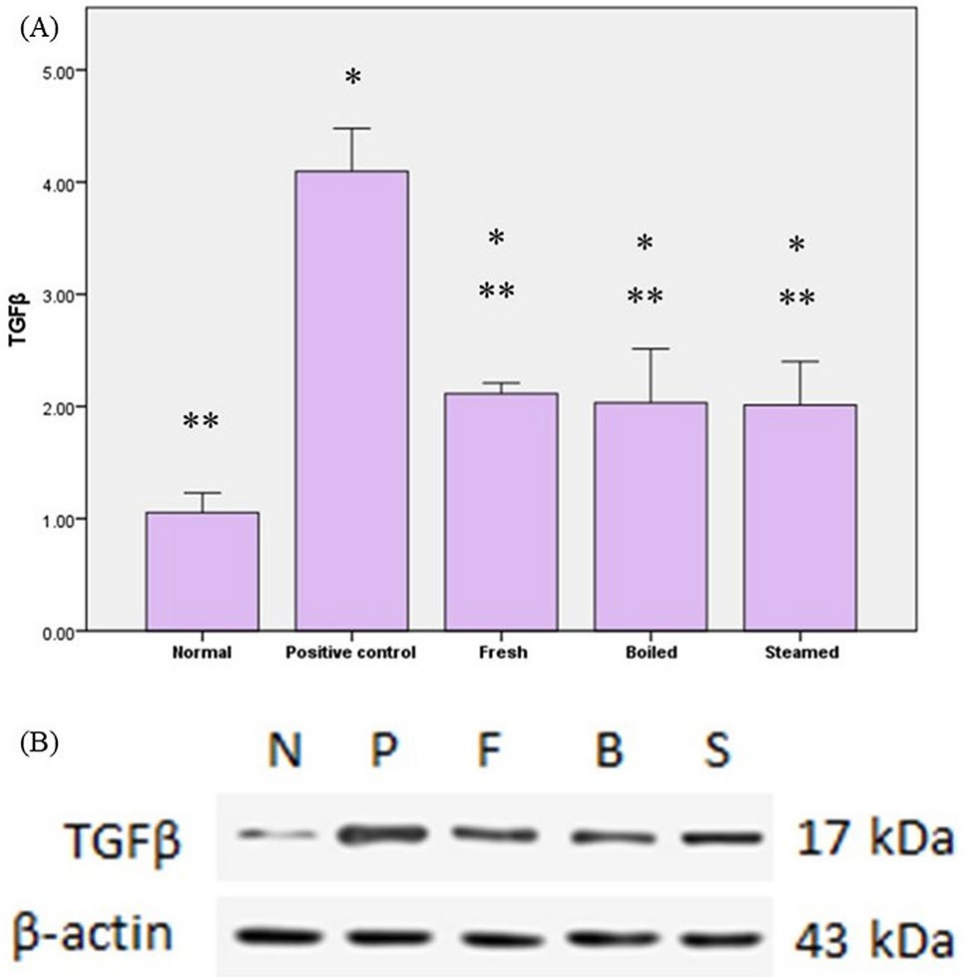
The impact of beetroot extracts on (**a**) tumor growth factor beta (TGF-β) levels in rats developing atherosclerosis due to a high-fat diet and (**b**) western blot. The data are presented as mean values with their respective standard deviations (SD). The statistical analysis included utilizing one-way ANOVA and Tukey’s post hoc test. * indicates statistically significant differences between groups at *P* < 0.05.

The levels of TGF-*β* were significantly raised in rats fed with an HFD by 288.4% when compared to normal. The pretreatment with varying beetroot extracts significantly downregulated the TGF-*β* expression by 48.3% in fresh extract, 50.3% in boiled extract, and 50.8% in steamed extract compared to the positive control, as demonstrated in Fig. 5.

### Light microscopic observations

**Fig. 6:**
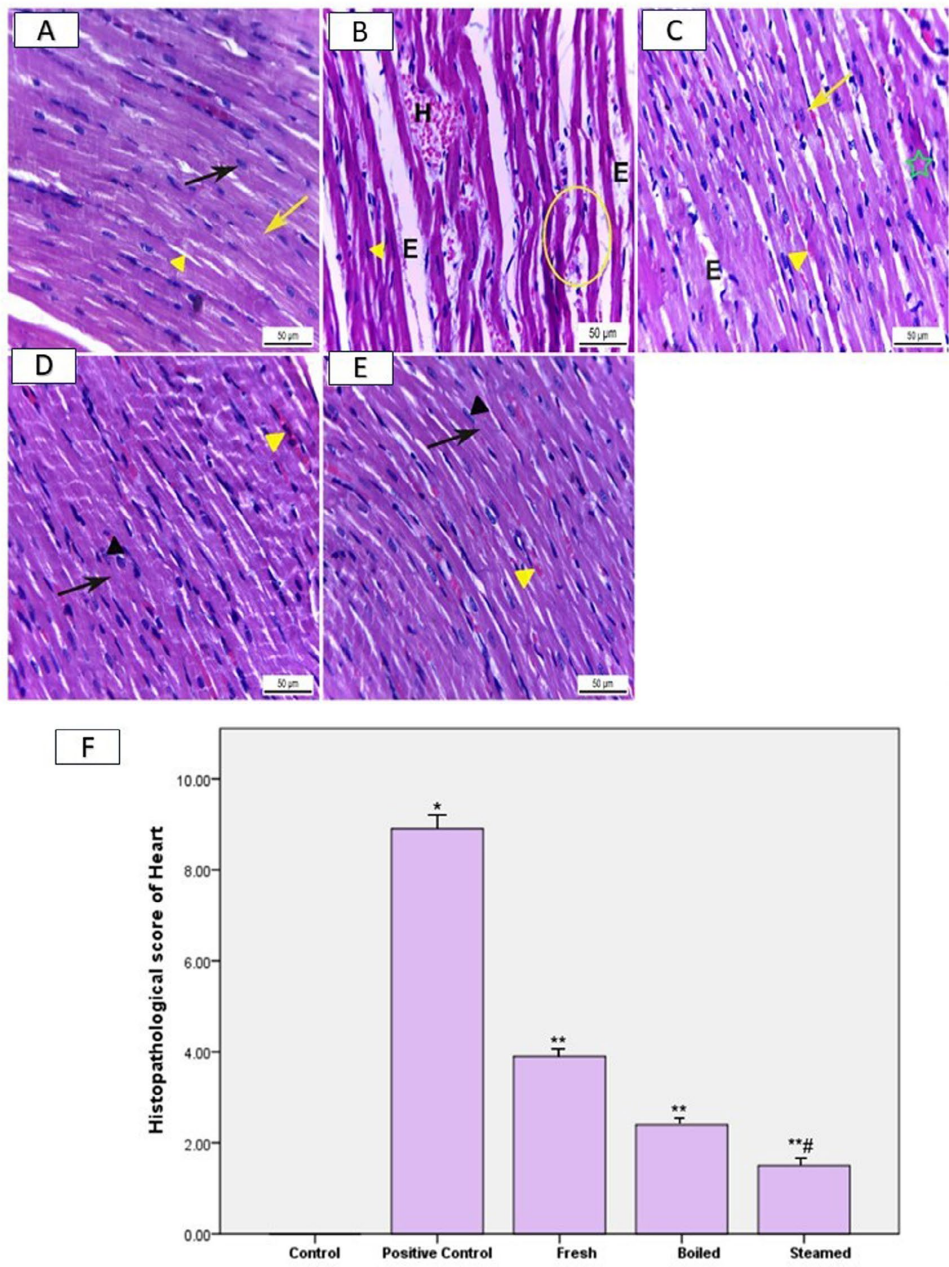
Heart sections of male Swiss albino rats. H&E.X400. (**A**) Negative control rats had normal elongated and branched cardiac muscle fibers (yellow arrow) with oval central nuclei (black arrow). There were interstices (yellow arrowhead) between the muscle fibers containing loose connective tissue (**B**) Positive control rats fed on an HFD showed hemorrhage (H), edema (E) between muscle fibers, and degenerated cardiac muscle fibers (circle) with flat pyknotic nuclei (yellow arrowhead). (**C**) Rats pretreated with fresh beetroot extract and then fed on HFD revealed decreased hemorrhage (yellow arrowhead) and edema (E); most cardiac muscle fibers appeared regular and nearly normal with oval central nuclei (yellow arrow) except a few degenerated muscle fibers (green star). Rats pretreated with boiled (**D**) and steamed (**E**) beetroot extracts, respectively, then fed on HFD demonstrated marked maintenance of the normal histological structure of heart muscle fibers versus the HFD group that appeared elongated branched (black arrows) with oval and central nuclei (black arrowhead) except for few hemorrhages (yellow arrowhead). F) Heart histopathological scores. The results are presented as mean ± SD. * indicates statistical significance compared to the control group, while ** denotes significance compared to the HFD group. A significance level of *P* ≤ 0.05 was used.

Heart sections of normal rats had the normal histological structure of cardiac muscle fibers. Each muscle fiber appeared elongated, branched, and cross-striated (yellow arrow) with a central oval nucleus (black arrow). Between muscle fibers were interstices (yellow arrowhead) containing loose connective tissue and blood capillaries (Fig. 6A). Conversely, heart sections obtained from positive control rats that fed on HFD showed hemorrhage (H), edema (E) between muscle fibers, and degeneration of cardiac muscle fibers (circle) that appeared separated from each other with pyknotic flat nuclei (yellow arrowhead) (Fig. 6B). In contrast, cardiac sections of rats pretreated with fresh beetroot extract and then fed on an HFD revealed a significant decrease in the histopathological alterations compared to a positive control group in the form of diminished hemorrhage (yellow arrowhead) and edema (E) between muscle fibers, most of the cardiac muscle fibers appeared regular and nearly normal with central oval nuclei (yellow arrow). However, few muscle fibers appeared degenerated (green star) (Fig. 6C). Furthermore, the groups administered steamed and boiled beetroot extracts exhibited marked maintenance of the normal histological structure of heart muscle fibers that appeared elongated and branched (black arrows) with oval and central nuclei (black arrowhead), except for a few hemorrhages (yellow arrowhead) observed (Fig. 6D and E).

### Histopathological score evaluations

The heart histopathological score was significantly (*P* ≤ 0.05) higher in rats fed HFDs than in negative control rats. However, the score was significantly reduced in rats pretreated with fresh, boiled, and steamed beetroot extracts compared to rats fed on HFD (Fig. 6F).

### Immunohistochemical assessment

**Fig. 7:**
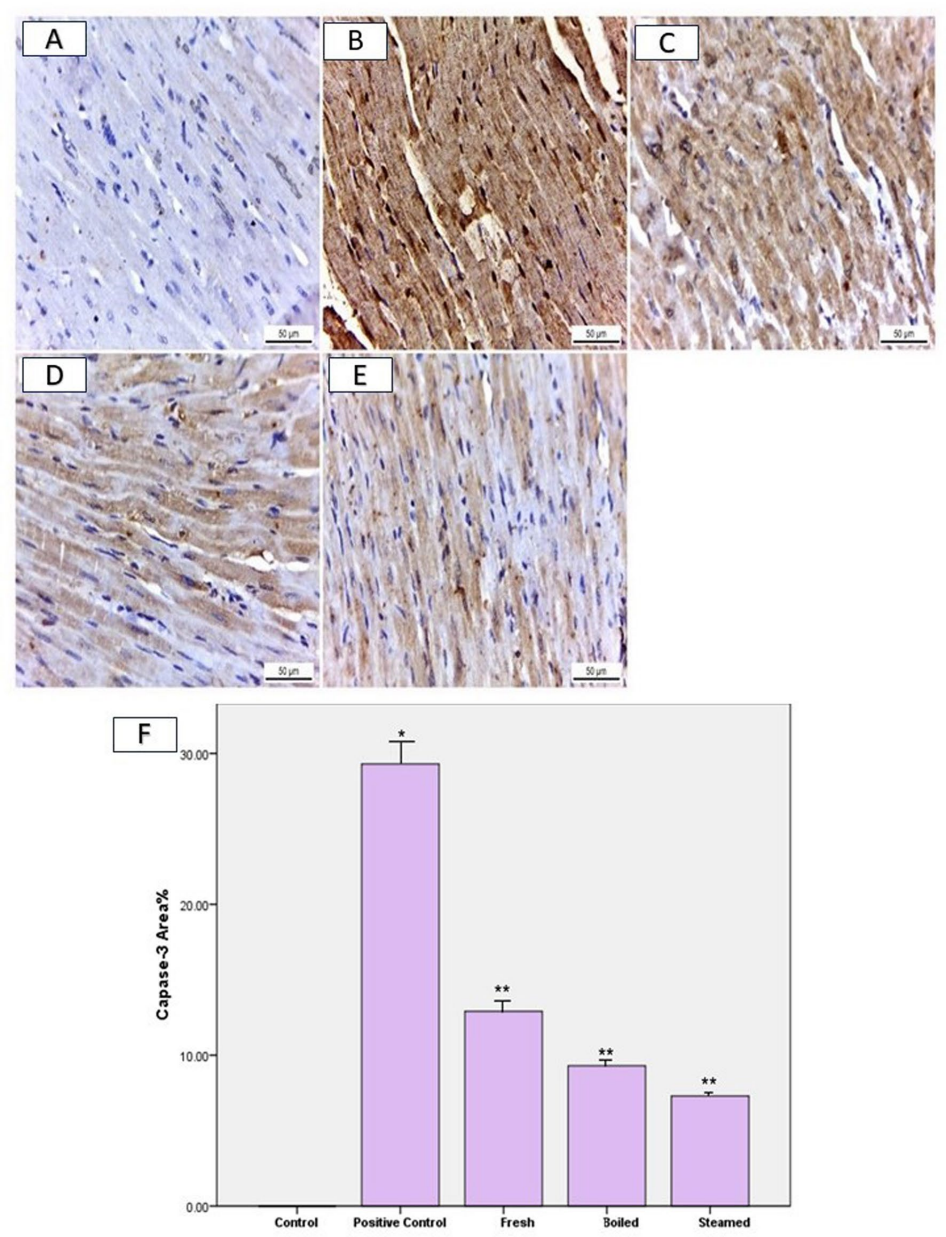
Immunohistochemical staining of Caspase-3 in heart sections at 400x magnification. (**A**) Negative caspase-3 expression in cardiac muscle fibers of normal control rats. (**B**) Strong positive caspase-3 immunoreactivity in positive control rats fed an HFD compared to the negative control group. (**C**) Rats pretreated with fresh beetroot extract followed by HFD showed moderate caspase-3 immunoreactivity versus the HFD group. (**D**) Heart sections from rats pretreated with boiled beetroot extract and (**E**) steamed beetroot extract before HFD displayed mild caspase-3 immunoexpression compared to the HFD group. (**F**) Percent area covered by caspase-3 positive immunoreactive cells within the rat heart, indicating the effect of HFD and pretreatment with fresh, boiled, and steamed beetroot extracts. * Significantly different from the control group. ** Significantly different from the HFD group. P value ≤ 0.05.

Caspase-3 staining in the heart sections of negative control rats exhibited no immunoreactivity within the cardiac muscle fibers (Fig. 7A). Conversely, rats fed an HFD displayed notably intense positive immunoreactivity of 29.3, significantly higher than the negative control group (Fig. 7B and F). In contrast, the caspase-3-stained heart sections of rats pretreated with fresh beetroot extract, then fed on HFD exhibited moderate immunoexpression significantly reduced by 12.9 compared to the HFD group (Fig. 7C and F). Furthermore, rats pretreated with steamed and boiled beetroot extracts and then fed on HFD showed mild caspase-3 immunoreactivity in cardiac muscles that significantly decreased by 9.3 and 7.3, respectively, compared to the HFD group (Fig. 7D, E, and F).

## Discussion

The current study was conducted to compare and discover the phytochemical composition of the fresh and processed beetroot extracts and their impact on cardiovascular protective activity in an obesity-induced rat model. The fresh, steamed, and boiled extracts were administered to the experimental groups for two weeks before the induction of obesity using HFD for an additional four weeks as an alternative animal model for obesity simulating the human syndrome.

Recent clinical data has revealed a significant association between obesity and cardiovascular complications^54,55^, as obesity can accelerate the incidence of different cardiovascular diseases and elevate cardiovascular morbidity and mortality risk.

Furthermore, recent studies on rats have highlighted the cardiac dysfunction and fibrotic effect of an HFD. These findings are consistent with the current study, which revealed elevated proinflammatory biomarkers and lipid peroxidation levels. Moreover, catalase serum activity was significantly reduced. Feriani et al. (2020) provided evidence that the significantly increased expression of cardiac TGF-β can account for the profibrotic effects of HFD^56^. Furthermore, the association between the activation of the TGF-β pathway and the development of cardiac fibrosis is widely recognized^57^. TGF-1 has been identified as an inductor of ECM synthesis, and it functions via the Smad gene^58^. These findings are consistent with recent studies investigating the role of the TGF-1/Smad signaling pathway in fibrotic events in obese animals^56^.

Furthermore, this is the first study to look at the potential fibrotic changes in heart tissues in animals fed an HFD and the possible protective effect of different processed red beetroot extracts. As a result, the current study suggests that HFD may exacerbate cardiovascular structural and functional changes. Interestingly, when different red beetroots extracts were used prior to the administration of the HFD, the other biomarkers were modulated and nearly restored to normal levels. The potential cardiovascular protective effect of the beetroot extracts, particularly the steamed extract, was indicated by the perfectly normal level of TNF-α, which was achieved by mitigating proinflammatory biomarkers, reducing oxidative stress, and profibrotic growth factors.

Evidence suggests that red beetroots enhance exercise performance, modulate rat insulin levels, and reduce oxidative stress^59,60^. However, a recent study demonstrated that daily consumption of concentrated red beetroot juice had no effect on glycemic control or blood pressure in patients compared with controls. This finding can be attributed to the various methods used to prepare the juice, the relatively short time it takes to observe such an effect or the necessity for additional assessment of oxidative and inflammatory biomarkers^61^.

In this research, heart sections obtained from rats fed on HFD revealed several histopathological alterations that agreed with the findings revealed by Rasheed and colleagues^62^. Additionally, our findings align with Dawood and Hareedy^63^, who illustrated that many muscle fibers in hyperlipidemic rats were lost with the marked widening of the intercellular spaces. Cardiac muscle damage may be due to the adverse effect of dyslipidemia at molecular and cellular levels^62^, which could also disrupt the antioxidant enzyme function, resulting in antioxidant/oxidant imbalance^64^.

On the other hand, rats pretreated with fresh, boiled, and steamed beetroot extracts showed ascending levels of maintenance of the almost normal histological architecture of cardiac muscle fibers, respectively. As the alterations disappeared gradually in the form of diminished hemorrhage and edema, most cardiac muscle fibers appeared regularly elongated and branched with oval central nuclei except for a few degenerated fibers. These findings agree with previous results, indicating that rats pretreated with beetroot juice and then injected with doxorubicin significantly maintained the normal histological morphology of myocardium^65^. *Beta vulgaris* (beetroots) contain important bioactive agents like betalains, polyphenols, and flavonoids, which have several physiological effects. Therefore, the chemical structure of beetroots refers to their antioxidant components that may be a cause of their protective role^66,67^.

Immunohistochemically, caspase-3 is strongly expressed in the myocardium of rats fed on an HFD compared to negative control rats. Meanwhile, the expression of caspase-3 decreased gradually in hyperlipidemic rats pretreated with steamed and boiled beetroot extracts, respectively, compared to the HFD group. These results indicate the anti-apoptotic effect of beetroots extracts.

The UPLC-QTOF-MS/MS analysis of fresh and processed extracts of beetroot revealed the predominance of polyphenolic compounds (flavonoid and phenolic acids), betacyanins, saponins, and organic acids, which could play a crucial role in the cardiovascular protective effects demonstrated in our study. Phenolics, flavonoids, glycosidic derivatives, and saponins are the most reported potential phytochemicals with significant cardioprotective effects, mainly when found together by acting with synergistic mechanisms^68^.

Betacyanins are an essential group of compounds with radical scavenging and antioxidant activities. They prevent oxidative processes that contribute to the onset of several degenerative disorders. Betanins exhibit high bioavailability and protect against certain stress-related oxidative disorders. In addition, they are characterized by numerous biological activities, including antioxidant, anti-inflammatory, lowering blood lipids, immune regulatory, hepatoprotective, and anticancer activities^2^. The concentration of red pigment (betacyanins) in red beetroots was approximately eight times higher than the yellow pigment (betaxanthins), while the difference was negligible in the case of their juice^40^. Beetroot pigments comprise more than 80% betacyanins, mainly betanin and its isomer, isobetanin. Due to the C15 chiral center, Betacyanins exist in two epimeric forms, e.g., betanin and isobetanin. Such epimerization led to an increased isobetanin concentration^40^. Processing results in about a 3-fold decrease in red pigment content. This finding can be attributed to betalains degradation that occurs during storage or processing. Betacyanins undergo degradation processes, including decarboxylation and glycoside elimination, resulting in the formation of yellow products such as neobetacyanins, betalamic acid, and betaxanthins^69,70^. This result is consistent with findings in the literature that indicate color alterations occur during the process of heating and storage^45^.

If saponin glycosides are subjected to moderate heat processing, they can be utilized in obese mice to achieve significant outcomes in inhibiting obesity lipid-lowering^71^. Saponins can induce cardioprotection against ischemia-reperfusion injury through bradykinin-NO pathway activation, followed by the suppression of free radicals and reactive oxygen species^68^. The reason for this is that the glycosides are present in both fresh and steamed specimens, especially steamed ones.

Early research has shown that steam and hot liquid water rapidly expand and break down the structure of the cell walls in the biomass. This facilitates the release of bioactive components from the plants and enhances biological activity and extraction efficiency. Furthermore, the steam explosion is a valuable method for releasing bound phenols from cell vacuoles by breaking the ester bonds that bind phenolics and polysaccharides in the cell wall matrix^72,73^.

## Conclusion

Beta vulgaris L. was subjected to thermal treatments (steaming and boiling) and examined using UPLC-QTOF-MS/MS and chemometric techniques for cardiovascular protective properties in this study. Food processing was found to enhance taste and extend shelf life, in addition to preserving beneficial phytochemicals. UPLC-QTOF-MS/MS led to the identification 51 metabolites, including phenolic acids, flavonoids, and saponins. PCA analysis revealed distinct profiles for fresh and processed samples. Steaming was found to be the optimal method, retaining phytochemicals and biological activity. In a hyperlipidemia and obesity rat model, different B. vulgaris L. extracts showed varying cardiovascular protective effects. Steaming resulted in the highest preservation of bioactive compounds, indicating superior effectiveness compared to fresh and boiled extracts.

### Study limitations and future prospectives

This study focused only on evaluating the efficacy of variously processed *Beta vulgaris* extracts, which limited the ability to examine these extracts on healthy rats alongside obese ones. Future research should investigate the impact of each extraction method on healthy rats as controls. Additionally, a comparative analysis of the steamed extract against other traditional cardioprotective drugs would provide valuable insights into its relative efficacy.

## Electronic Supplementary Material

Below is the link to the electronic supplementary material.

Supplementary Material 1

Supplementary Material 2

## Data Availability

The authors declare that the data supporting the findings of this study are available within the paper.
