# Supplementary figures and images for: Impact of thermal processing on phytochemical profile and cardiovascular protection of Beta vulgaris L. in hyperlipidemic rats
Source: Sci Rep. 2024 Nov 11;14:27539. doi: 10.1038/s41598-024-77860-2 (PMC11554672; doi:10.1038/s41598-024-77860-2)

| Biomarker | Original Image                                                                      | Molecular Weight |
|-----------|-------------------------------------------------------------------------------------|------------------|
| B-actin   | 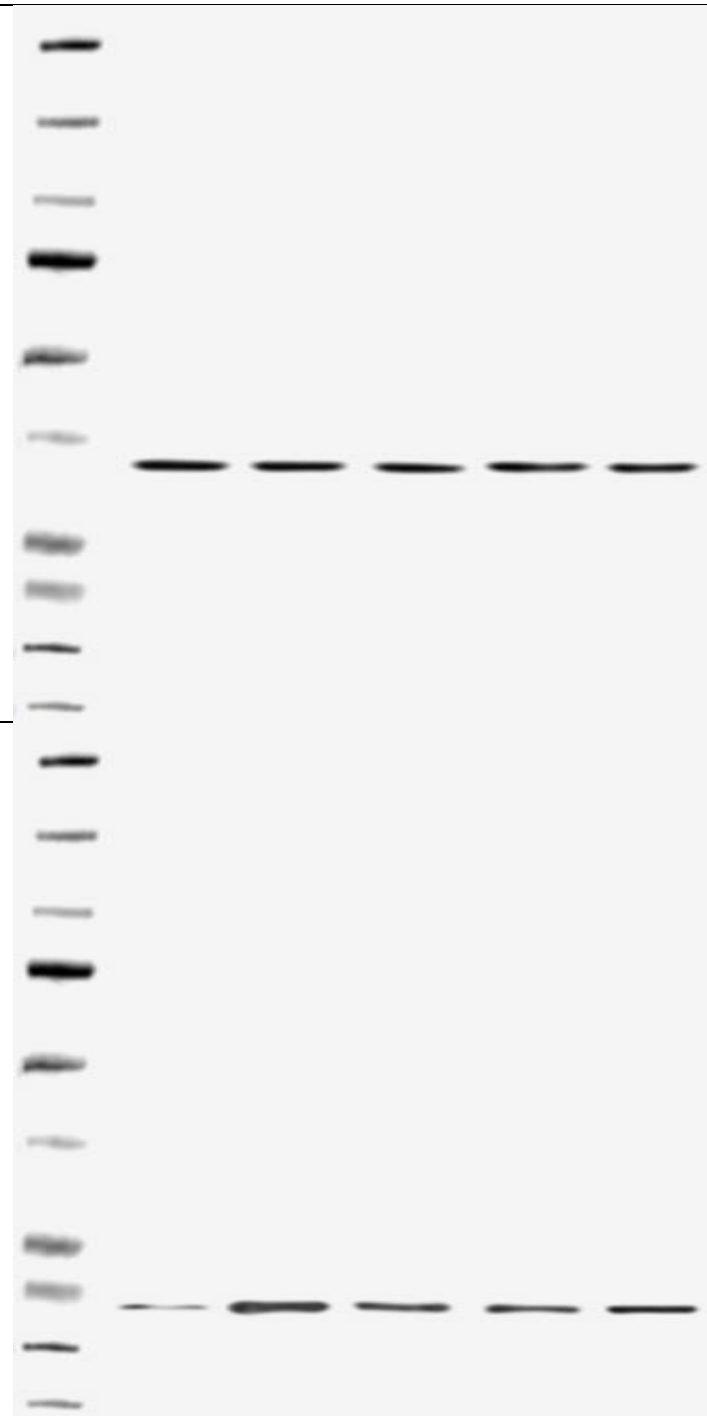 | 43 KDa           |
| TGF-β     |                                                                                     | 17 KDa           |

Supplement: Supplementary file 1 — Supplementary Material 1 [file 41598_2024_77860_MOESM1_ESM.pdf]
