# Supplementary material for: Impact of thermal processing on phytochemical profile and cardiovascular protection of Beta vulgaris L. in hyperlipidemic rats
Source: Sci Rep. 2024 Nov 11;14:27539. doi: 10.1038/s41598-024-77860-2 (PMC11554672; doi:10.1038/s41598-024-77860-2)
Supplement: Supplementary file 2 — Supplementary Material 2 [file 41598_2024_77860_MOESM2_ESM.docx]

| **Groups** | **MDA (nmol/ml)** | **Catalase (nmol/ml)** | **TNF-α (pg/ml)** | **IL-6 (pg/ml)** | **TGF-β** |
| --- | --- | --- | --- | --- | --- |
| Normal | 0.285 | 2.27 | 18.4 | 131.7 | 1 |
|  | 0.331 | 1.78 | 22.5 | 122.4 | 1.007186 |
|  | 0.172 | 1.92 | 27.5 | 128.4 | 1.156488 |
| Positive | 0.945 | 0.673 | 184.3 | 725 | 4.278565 |
|  | 0.896 | 0.852 | 146.5 | 817.4 | 3.896655 |
|  | 1.156 | 0.483 | 177.2 | 622.8 | 4.114535 |
| Fresh Extract | 0.674 | 1.52 | 117.3 | 257.5 | 2.165454 |
|  | 0.428 | 1.68 | 103.8 | 262.3 | 2.071679 |
|  | 0.631 | 1.57 | 66.7 | 277.3 | 2.108786 |
| Boiled Extract | 0.575 | 2.1 | 105 | 246.8 | 2.001655 |
|  | 0.443 | 1.83 | 77.4 | 252.5 | 1.808977 |
|  | 0.581 | 1.43 | 53.2 | 243.6 | 2.287676 |
| Steamed Extract | 0.387 | 1.95 | 67.4 | 238.7 | 2.138797 |
|  | 0.566 | 1.77 | 43.2 | 255.2 | 2.109866 |
|  | 0.365 | 1.65 | 55.3 | 247.3 | 1.789768 |

Table S1: Biochemical parameters assessed in the manuscript
